# Supplementary material for: MCP1 Could Mediate FGF23 and Omega 6/Omega 3 Correlation Inversion in CKD
Source: J Clin Med. 2022 Nov 30;11(23):7099. doi: 10.3390/jcm11237099 (PMC9739884; doi:10.3390/jcm11237099)
Supplement: Supplementary file 1 [file jcm-11-07099-s001.zip › jcm-2021507-supplementary.pdf]

Table S1: Correlation between PUFA and biochemical markers among CKD stage 3

CKD3	eGFR		MCP−1 (pg/mL)		iFGF23 (pg/mL)		cFGF23 (RU/mL)	
	r^2	p-value	r^2	p-value	r^2	p-value	r^2	p-value
PUFA	−0.010	0.968	0.422	0.092	0.116	0.637	−0.015	0.952
**n-3**	−0.032	0.897	0.162	0.535	**0.456**	**0.050**	0.071	0.772
18:3n3	−0.101	0.680	−0.209	0.421	0.145	0.554	0.161	0.510
20:5n3	−0.103	0.675	0.130	0.619	0.297	0.218	0.234	0.334
**22:5n3**	−0.114	0.642	−0.020	0.940	**0.628**	**0.004**	0.076	0.758
22:6n3	0.002	0.994	0.223	0.390	0.325	0.175	−0.107	0.663
n-6	0.171	0.483	0.377	0.135	−0.056	0.819	−0.267	0.270
18:2n6	0.201	0.410	0.270	0.295	−0.158	0.519	−0.055	0.822
18:3n6	0.052	0.833	−0.267	0.299	−0.054	0.828	0.295	0.221
20:3n6	0.217	0.371	−0.304	0.236	0.211	0.387	−0.163	0.504
20:4n6	−0.153	0.533	0.377	0.135	−0.056	0.819	−0.376	0.112
22.4n6	0.203	0.404	−0.347	0.173	0.181	0.457	0.096	0.696
22:5n6	0.328	0.170	0.062	0.814	−0.009	0.971	−0.136	0.580

The values in green indicate a significant positive correlation. "−" indicates a negative correlation. n=19.

Table S2: Correlation between PUFA and biochemical markers among CKD stage 4

CKD4	eGFR		MCP-1 (pg/mL)		iFGF23 (pg/mL)		cFGF23 (RU/mL)	
	r^2	p-value	r^2	p-value	r^2	p-value	r^2	p-value
PUFA	0.043	0.839	0.147	0.493	−0.073	0.734	−0.204	0.339
n-3	−0.255	0.219	−0.032	0.881	0.092	0.670	0.244	0.251
18:3n3	0.285	0.167	−0.256	0.228	0.207	0.331	0.247	0.245
20:5n3	−0.119	0.570	−0.012	0.955	0.114	0.594	0.380	0.067
22:5n3	−0.201	0.336	0.325	0.122	−0.191	0.371	0.154	0.473
**22:6n3**	**−0.414**	**0.040**	−0.056	0.796	0.182	0.396	0.125	0.561
n-6	0.119	0.572	0.120	0.576	−0.132	0.539	−0.253	0.233
18:2n6	0.131	0.531	−0.134	0.533	−0.022	0.920	−0.260	0.219
18:3n6	0.003	0.990	0.187	0.381	−0.250	0.240	−0.005	0.980
**20:3n6**	−0.202	0.333	**0.594**	**0.002**	−0.133	0.537	0.247	0.245
**20:4n6**	−0.194	0.352	**0.424**	**0.039**	−0.221	0.300	−0.142	0.507
22.4n6	−0.107	0.611	0.344	0.099	−0.134	0.532	0.002	0.994
**22:5n6**	−0.335	0.102	**0.521**	**0.009**	−0.081	0.707	0.153	0.474

The values in red/green indicate a significant negative/positive correlation. "−" indicates a negative correlation. n=25.

Table S3: Correlation between PUFA and biochemical markers among CKD stage 5

**CKD5**	**eGFR**		**MCP-1 (pg/mL)**		**iFGF23 (pg/mL)**		**cFGF23 (RU/mL)**	
	r^2	p-value	r^2	p-value	r^2	p-value	r^2	p-value
PUFA	−0.178	0.580	−0.231	0.471	−0.077	0.812	−0.119	0.713
**n-3**	0.134	0.677	−0.280	0.379	−0.462	0.131	**−0.587**	**0.045**
18:3n3	0.323	0.305	0.210	0.513	−0.196	0.542	−0.322	0.308
20:5n3	0.124	0.702	0.119	0.713	−0.469	0.124	−0.280	0.379
22:5n3	−0.147	0.648	0.056	0.863	−0.165	0.609	0.077	0.812
**22:6n3**	0.217	0.499	−0.326	0.301	−0.476	0.117	**−0.627**	**0.029**
n-6	−0.069	0.831	−0.182	0.572	−0.007	0.983	0.119	0.713
18:2n6	−0.389	0.212	−0.077	0.812	0.203	0.527	−0.070	0.829
**18:3n6**	−0.510	0.090	**0.677**	**0.016**	0.140	0.664	0.133	0.680
20:3n6	0.414	0.181	0.371	0.236	−0.056	0.863	0.301	0.342
20:4n6	0.189	0.557	0.007	0.983	−0.531	0.075	0.301	0.342
22.4n6	0.096	0.768	0.092	0.776	−0.188	0.559	0.428	0.165
**22:5n6**	0.275	0.387	0.158	0.624	−0.081	0.803	**0.632**	**0.028**

The values in red/green indicate a significant negative/positive correlation. "−" indicates a negative correlation. n=12.
